# The Association Between Oral Health and the Tendencies to Obsessive–Compulsive Behavior in Biomedical Students—A Questionnaire Based Study

**DOI:** 10.3390/medicina61040593

**Published:** 2025-03-26

**Authors:** Dinko Martinovic, Matea Cernak, Slaven Lasic, Ema Puizina, Antonella Lesin, Mihaela Rakusic, Marino Lupi-Ferandin, Laura Jurina, Ena Kulis, Josko Bozic

**Affiliations:** 1 Department of Maxillofacial Surgery, University Hospital of Split, 21000 Split, Croatia; dmartinovic@kbsplit.hr (D.M.);; 2 Study of Dental Medicine, University of Split School of Medicine, 21000 Split, Croatia; 3 Department of Neurology, University Hospital Dubrava, 10000 Zagreb, Croatia; 4 Department of Psychiatry, University Hospital of Split, 21000 Split, Croatia; 5 Department of Pathophysiology, University of Split School of Medicine, 21000 Split, Croatia

**Keywords:** biomedical students, obsessive–compulsive personality disorder, perfectionism, oral health

## Abstract

*Background and Objectives:* Oral health is a critical component of overall well-being, and it is highly influenced by psychological factors. While mental health disorders are often associated with poor oral health, the impact of obsessive–compulsive personality disorder (OCPD) tendencies on oral hygiene behaviors remains underexplored. This study aimed to investigate the relationship between OCPD tendencies and oral health behaviors among biomedical students. *Materials and Methods:* A cross-sectional survey was conducted among 384 biomedical students aged 18–30 years. Participants completed the Hiroshima University Dental Behavior Inventory (HU-DBI) to assess oral health behaviors and the Five Factor Obsessive–Compulsive Inventory—Short Form (FFOCI-SF) to evaluate OCPD tendencies, and they were divided into high and low tendencies groups. Sociodemographic data were also collected. Statistical analysis of all collected data was performed using the MedCalc computer software for Windows^®^. *Results:* The higher OCPD tendencies group was older (*p* = 0.003), with predominantly female students (*p* = 0.005), and with higher academic grades (*p* < 0.001). These participants exhibited significantly better HU-DBI total scores compared to the group with the lower tendencies (8.0 (7.0–8.0) vs. 7.0 (6.0–8.0); *p* < 0.001). Traits such as perfectionism, fastidiousness, and punctiliousness were significantly associated with better oral hygiene practices (*p* < 0.001). *Conclusions:* Our results suggest that OCPD tendencies in biomedical students positively influence oral health behaviors through traits like perfectionism and attention to detail, though excessive rigidity may pose risks such as over-brushing. However, future longitudinal larger-scale studies need to address these findings.

## 1. Introduction

Recently oral health has gained a new and comprehensive definition published by the FDI World Dental Federation. What now constitutes oral health is the overall well-being of the mouth, teeth, gums, and related structures, encompassing the ability to perform essential functions such as speaking, smiling, chewing, swallowing, and conveying emotions through facial expressions with confidence and without discomfort, pain, or disease. It is recognized now that oral health exists in a continuum according to the values and attitudes of individuals and communities, reflecting physiological, social, and psychological attributes, and is essential for the overall quality of life [1,2,3]. The oral cavity is often referred to as the “window to health”, and an idea of the brain–stomatognathic axis provides more evidence that the bidirectional effect of oral health and somatic health can be translated also to psychiatric and neurological health [4]. Mental health disorders have a strong association with poor oral health and vice versa [4]. Disorders such as anxiety, depression, schizophrenia, dementia, and personality disorders are often associated with inadequate oral health, increased dental decay, as well as greater tooth loss and temporomandibular disorders [4,5,6].

Obsessive–compulsive personality disorder (OCPD) is a mental disorder categorized under Cluster C personality disorders according to DSM V [7]. It is a maladaptive chronic personality disorder characterized by excessive perfectionism and the need for control of the environment and oneself. Even though OCPD and OCD sometimes co-occur, with 25% of persons with OCD having OCPD, these are distinct mental disorders [7,8]. OCD is a mental disorder characterized by obsessions (recurring distressing ideas) and compulsions (recurring actions used to decrease anxiety caused by obsessions) categorized independently from anxiety disorders [7]. The difference is in the ego-syntonic symptoms of OCPD compared to the ego-dystonic symptoms of OCD; in other words, persons with OCPD exhibit their symptoms out of the need for perfection, order, and control while persons with OCD do it to alleviate the anxiety of obsessions [7,8,9]. OCPD constitutes eight personality traits: rigidity, perfectionism, over-attention to detail, miserliness, excessive devotion to work, hyper morality, inability to discard worn or useless items, and inability to delegate tasks [7,8,9]. Excessive preoccupations often permeate all aspects of the patient’s life, cause impairment in daily functioning, and could potentially lead to the development of secondary mental disorders [7]. It is a common disorder affecting around 2–8% of the global population, most often starting in early adulthood, and according to some studies, more prevalent in men [8]. Etiology is mixed with genetic, environmental, and neurobiological factors. It is believed that alterations in the dopaminergic and serotoninergic neurotransmitter system, alongside parental overcontrol and perfectionism, lead to the development of OCPD [8,9]. The diagnosis of OCPD can be made using clinical assessments based on the Diagnostic and Statistical Manual of Mental Disorders (DSM-V-TR). Integrating this assessment tool with clinical judgment, collateral information, and a thorough history is mandatory [10]. However, OCPD maladaptive personality traits can also be explored using a dimensional trait model.

OCPD characteristics could have a twofold effect on oral health. Perfectionism, attention to detail, and need for control could lead to meticulous oral care routines, healthy diet, and physical exercise in persons with OCPD tendency, which would lead to good oral health. However, rigidity and perfectionism could lead to over-brushing or flossing leading to enamel erosion, gum bleeding, and recession, while anxiety co-occurring with OCPD could lead to teeth grinding or clenching resulting in tooth wear and jaw pain. To the best of their knowledge, the authors found no data on how OCPD characteristics translate to oral health. Also, only one study reported on the effect of OCD on oral health habits and oral health with results showing that the unique aspects of the disorder, such as obsessions, potentially lead to better brushing habits, leading to lower caries experience compared to the control group [11].

The goal of this cross-sectional study was to examine the oral health habits of biomedical students and further investigate whether there is a possible association with OCPD tendencies.

## 2. Materials and Methods

### 2.1. Study Design

This cross-sectional questionnaire-based study was conducted continuously at the University of Split School of Medicine during the period from July 2023 to January 2024.

All respondents were informed in advance about the purpose and goals of the research, ensuring transparency and voluntary participation. The completion and submission of the survey questionnaire were considered as providing informed consent to participate in this study.

The research methodology adhered strictly to ethical standards and was approved by the Ethics Committee of the Faculty of Medicine in Split (Class: 003-08/23-03/0015; Reg. Number: 2181-198-03-04-23-0056). This study was conducted following the latest guidelines outlined in the Declaration of Helsinki (2013), ensuring that participants’ rights were preserved throughout the research process.

### 2.2. Subjects

The research utilized a structured questionnaire created and distributed through the Google Forms^®^ (Google LCC, Mountain View, CA, USA) application, which allowed for easy access and completion by participants. The link to the questionnaire was shared among students via multiple communication channels, including direct messages, emails, and through student representatives and student groups on social media platforms. This approach ensured that a wide range of students had the opportunity to participate in the study. Participation was entirely voluntary, with anonymity guaranteed to encourage honest and unbiased responses.

The inclusion criteria for participation were as follows: students aged between 18 and 30 who were enrolled in biomedical sciences programs at the University of Split School of Medicine. Individuals with diagnosed psychiatric conditions and those who submitted incompletely filled questionnaires were excluded from the final analysis to maintain data quality and reliability.

A total of 399 students initially completed the questionnaire, resulting in a response rate of 33.4%. However, after reviewing submissions for completeness, 15 respondents were excluded due to incomplete questionnaires. Consequently, the final number of participants included in the analysis was 384. The exclusion process ensured that only high-quality data were used for statistical analysis, enhancing the validity of the study’s findings.

### 2.3. Questionnaires

The conducted survey consisted of three distinct parts, each designed to gather specific data relevant to the study’s objectives. This ensured that the collected data were comprehensive and allowed for the in-depth analysis of sociodemographic factors, oral health behaviors, and personality traits.

The first part focused on sociodemographic data, aiming to provide a comprehensive overview of the participants’ backgrounds. This section included questions about gender, age, field of study, year of study, and average academic grade. Additionally, participants were asked about their economic situation, categorized into three levels: low, average, and high. This information helped contextualize the findings within socioeconomic frameworks. Furthermore, respondents were queried about any prior known psychiatric diseases or conditions. This aspect was essential for identifying potential confounding variables and to exclude these participants.

The second part of the survey employed the Hiroshima University Dental Behavior Inventory (HU-DBI), a validated and reliable instrument used to assess oral health behavior. The HU-DBI consists of 20 statements with dichotomous answers (agree/disagree). Of these, 12 questions are scored with either 0 or 1 points depending on the response, while the remaining 8 questions are not scored [12]. The total score ranges from 0 to 12, with higher scores indicating better oral health care practices. The HU-DBI also allows for further analysis by dividing the results into three subdimensions: knowledge, attitudes, and behavior. These subdimensions provide a more nuanced understanding of participants’ oral health practices and their underlying motivations or barriers.

The third part utilized the Five-Factor Obsessive–Compulsive Inventory—Short Form (FFOCI-SF), a validated and reliable tool designed to examine personality traits associated with OCPD [13]. This questionnaire comprises 48 statements rated on a 5-point Likert scale ranging from “strongly disagree” to “strongly agree”. Each response is scored from 1 to 5 points, with higher total scores indicating a stronger tendency toward OCPD traits. The FFOCI-SF also measures several distinct dimensions of OCPD, including excessive worry, detached coldness, risk aversion, constricted behavior, inflexibility, dogmatism, perfectionism, fastidiousness, punctiliousness, workaholic tendencies, doggedness, and ruminative deliberation. By examining these dimensions individually and collectively, the instrument provides a detailed profile of personality traits that may influence behaviors or attitudes relevant to the study.

### 2.4. Statistical Analysis

The required sample size for this study was determined using the Surveymonkey^®^ online calculator. The target population consisted of 1194 biomedical students at the University of Split. We aimed for a 95% confidence interval and a 5% margin of error, which is commonly used in survey-based research to ensure a high degree of certainty in the results while balancing precision and practicality. According to the calculator, the required sample size was estimated at 291 subjects.

Statistical analysis of all collected data was conducted using MedCalc computer software for Windows^®^ (MedCalc Software, Ostend, Belgium, version 23.16). Additionally, all graphical figures were created using SigmaPlot software for Windows^®^ (version 15.0, Systat Software Inc., San Jose, CA, USA), which provides advanced capabilities for visually representing complex data in a clear and interpretable manner.

Categorical variables were summarized as whole numbers and percentages to provide a clear overview of the distribution within the dataset. Quantitative variables were presented based on the normality of their distribution: normally distributed data were expressed as arithmetic mean ± standard deviation (SD), while non-normally distributed data were presented as median with interquartile range (IQR). The normality of the distribution was evaluated using the Kolmogorov–Smirnov test, a standard method for assessing whether data follow a normal distribution. For statistical comparisons, categorical variables were analyzed using the Chi-square test or Fisher’s exact test, depending on sample size and expected frequencies. Quantitative variables were compared using either Student’s *t*-test for normally distributed data or the Mann–Whitney U test for non-normally distributed data. Correlations between variables were assessed using Spearman’s correlation test, which is particularly suitable for non-parametric data. The threshold for statistical significance was set at *p* < 0.05, ensuring that the results were interpreted with a high level of confidence.

## 3. Results

This study included 384 biomedical students, out of which 112 (29.2%) were male and 272 (70.8) were female. The mean age of the study population was 23.2 ± 1.9 years, and most of the included students were from medical studies 172 (44.8). Furthermore, most of the subjects had an average economic situation (70.3%) and most of them were in the 6th year of their studies (33.3%). The average grade in their studies was 4.0 (3.8–4.2), and most of them were not smokers (69.3%), while on the other hand, most of them consumed alcohol (57.8%).

Subjects were divided into a group with a higher tendency toward OCPD and a group with a lower tendency toward OCPD, depending on the median of the FFOCI-SF total score. The higher tendency group had an FFOCI-SF total score > 144, while the lower tendency group had an FFOCI-SF total score ≤ 144. When comparing the sociodemographic characteristics between the two groups, it was found that the group with a higher tendency toward OCPD had a significantly older age (*p* = 0.003) and that it had a significantly higher average grade at college (*p* < 0.001) (Table 1). Also, it was found that a significantly higher proportion (*p* = 0.005) of subjects from the group with a higher tendency toward OCPD were females (78.1%) when compared to the lower tendency group (64.6%) (Table 1).

The median total score of the HU-DBI questionnaire was 7.0 (6.0–8.0). Among the items that contribute to the total score (2, 4, 6, 8, 9, 10, 11, 12, 14, 15, 16, and 19), most of the students indicated better oral health by agreeing with the statement “I often check my teeth in a mirror after brushing” (79.7%) and agreeing with the statement “I brush each of my teeth carefully” (77.1%) (Table 2). Furthermore, most showed better oral health with disagreeing on the statements “My gum tends to bleed when I brush my teeth” (94.3%) and “I think that I cannot help having false teeth when I am old” (84.9%). On the other hand, most showed poor oral health by disagreeing with the statement “I have used a dye to see how clean my teeth are” (13.5%) and “I have noticed some white sticky deposits on my teeth” (6.2%).

**Figure 1:**
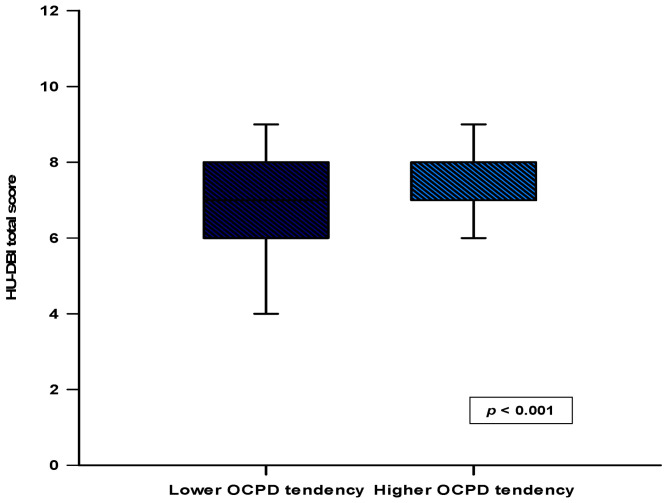
Comparison of the HU-DBI total score between the group with the lower tendency toward OCPD (FFOCI-SF ≤ 144) and the group with a higher tendency toward OCPD (FFOCI-SF > 144). Abbreviations: HU-DBI—The Hiroshima University Dental Behaviour Inventory.

**Figure 2:**
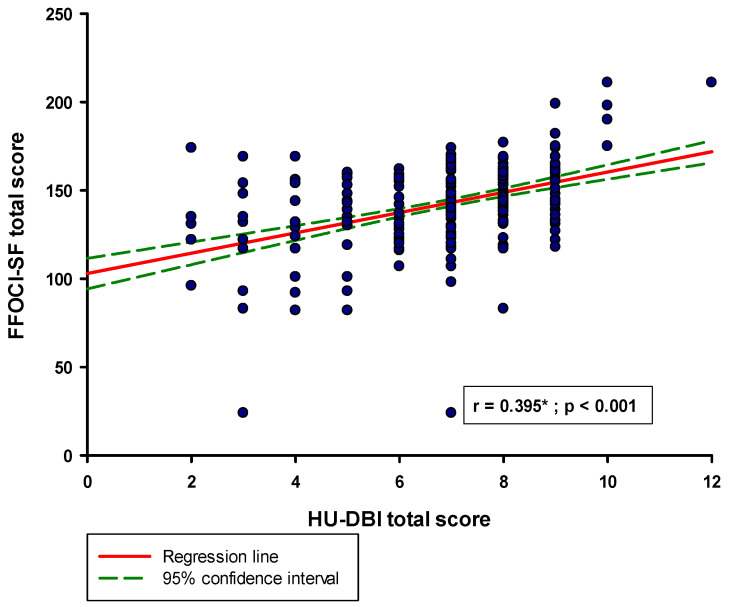
Correlation between the FFOCI-SF total score and HU-DBI total score (N = 384). Abbreviations: HU-DBI—The Hiroshima University Dental Behaviour Inventory; FFOCI-SF—Five Factor Obsessive–Compulsive Inventory—Short Form. * Spearman correlation coefficient.

The median score of the FFOCI-SF questionnaire was 144.0 (130.0–157.0). When comparing the total score of the HU-DBI questionnaire, it was found that the group with a higher tendency toward OCPD had a significantly higher score compared to the group with a lower tendency toward OCPD (8.0 (7.0–8.0) vs. 7.0 (6.0–8.0); *p* < 0.001) (Figure 1). Also, a statistically significant positive correlation was found between the total sum of HU-DBI and the total sum of FFOCI-SF (r = 0.395; *p* < 0.001) (Figure 2).

Furthermore, all three subdimensions showed significant positive correlation with the FFOCI-SF total score: knowledge (*p* < 0.001), attitudes (*p* < 0.001), and behavior (*p* < 0.001) (Table 3).

Most of the FFOCI-SF subdimensions showed a significant positive correlation with the HU-DBI total score: excessive worry (*p* < 0.001), perfectionism (*p* < 0.001), fastidiousness (*p* < 0.001), punctiliousness (*p* < 0.001), workaholism (*p* < 0.001), doggedness (*p* < 0.001), and ruminative deliberation (*p* < 0.001) (Table 4).

## 4. Discussion

The findings of this study demonstrated a significant positive correlation between FFOCI-SF results and HU-DBI results. Students with higher OCPD tendencies, as measured by the FFOCI-SF, exhibited better oral health practices, as reflected in higher HU-DBI scores. Specifically, the higher OCPD tendencies group had a median HU-DBI score of 8.0 compared to 6.0 in the lower OCPD tendencies group, with a significant positive correlation between FFOCI-SF and HU-DBI scores. These findings align with prior research suggesting that traits such as perfectionism and meticulousness promote adherence to structured routines, including oral hygiene practices [14]. Further analysis of the subdimensions provided additional insights into this relationship. Traits such as perfectionism, fastidiousness, and punctiliousness were significantly correlated with improved oral hygiene practices. This is consistent with previous studies indicating that perfectionism is a central feature of OCPD and contributes to disciplined health-related behaviors [15]. Furthermore, sociodemographic analysis revealed that students in the high-OCPD group were more likely to be older. Additionally, age was associated with higher HU-DBI scores, which aligns with studies showing that students in advanced academic years generally have elevated HU-DBI scores. These findings are consistent with previous studies suggesting that OCPD traits are more prevalent among individuals with high academic performance, but they are contrary to the fact that they are usually more prevalent among men [16]. The connection between perfectionism and academic success may explain why these traits extend to other domains, such as health maintenance.

Another interesting finding of this study was the significantly lower proportion of alcohol consumption among students with higher OCPD tendencies compared to those with lower tendencies. This observation aligns with the behavioral patterns of individuals with OCPD, who often avoid risky or unhealthy behaviors [17]. Alcohol consumption has been linked to poor oral health outcomes, including increased risks of periodontal disease, dental caries, and gum inflammation [18,19,20]. Chronic alcohol use alters the oral microbiome, reduces saliva production, and promotes acid-producing pathogens, all of which exacerbate oral health issues [21]. Therefore, the lower prevalence of alcohol use in the high-OCPD group may partly explain their superior oral health behaviors.

Dumitrescu et al. found that perfectionism, a primary trait of OCPD, positively impacts oral hygiene due to its association with self-esteem and self-image, which are closely tied to dental esthetics [22]. Oral hygiene affects the appearance of teeth, which significantly contributes to self-image. Another study demonstrated that perfectionism is more strongly associated with obsessive–compulsive personality symptoms than with OCD, and the pairing of perfectionism with OCPD is a relevant behavioral trait underlying vulnerability to eating disorders [23]. Additionally, a recent study showed that high perfectionism predicted higher self-perception of body image and lower mental health and self-esteem in college students, as well as better smile appearance.

The results also resonate with existing literature on OCD and oral health habits. While OCD and OCPD are distinct conditions, both share features such as heightened attention to detail and routine adherence. A recent study showed that individuals with OCD exhibit superior brushing habits and lower caries prevalence compared to controls [11]. However, unlike OCD behaviors, which are ego-dystonic and aimed at alleviating anxiety, OCPD behaviors are ego-syntonic and motivated by a desire for control and perfection [24]. Despite these positive associations, it is important to consider the potential risks associated with excessive rigidity and perfectionism in individuals with high OCPD tendencies. Excessive perfectionism may lead to maladaptive behaviors such as over-brushing or flossing, potentially resulting in enamel erosion or gum recession [23], while co-occurring anxiety disorders may result in bruxism (teeth grinding), causing tooth wear and jaw pain [25]. Duran and Sim presented a case of an OCD patient with oral lichen planus whose obsessive rituals increased its severity, demanding intensive pharmacological and cognitive behavioral therapy [26]. 

These risks highlight the need for balanced interventions that leverage the strengths of individuals with OCPD tendencies while addressing potential harms. The results of this study also underscore the broader implications of mental health on oral health outcomes. Mental disorders such as anxiety and depression have been associated with poor oral health due to the neglect of hygiene routines or the avoidance of dental visits caused by dental anxiety [27]. Conversely, poor oral health can exacerbate mental disorders through mechanisms such as chronic inflammation or social isolation resulting from tooth loss or malocclusion [4]. The bidirectional relationship between mental health and oral health highlights the necessity for integrated care approaches that address both physical and psychological well-being. People with mental disorders have difficulty voicing their concerns regarding oral health, following proper oral care routines, and experiencing dental anxiety, resulting in avoidance of dentists and causing a vicious cycle [4,5].

The findings of this study align with the biopsychosocial model of health, which emphasizes the interplay between biological, psychological, and social factors in determining overall well-being [28,29]. In this context, personality traits like those associated with OCPD can be seen as mediators influencing health behaviors. While traits such as perfectionism may promote positive habits like disciplined oral care routines, they can also lead to maladaptive outcomes if not managed appropriately. Health professionals should collaborate and engage with one another across oral and mental health care settings to improve the mental, oral, and overall health and well-being of this population.

On that note, it is worth comparing our study group’s HU-DBI scores with those from similar biomedical backgrounds. A study by Komabayashi et al. used the HU-DBI to assess oral health attitudes among British and Chinese dental students. Although the survey included only dental students, several noteworthy conclusions emerged compared to our study groups. Croatian students had a median score of 7.0, while British students averaged 7.33—significantly higher than the Chinese average of 5.07. Most students across all three countries exhibited good oral hygiene practices. For instance, 79.7% of Croatian students reported checking their teeth in a mirror after brushing, and 77.1% stated they brush each tooth carefully. Notably, 76% of British students and 63% of Chinese students felt they brushed carefully, with 72% regularly checking their teeth in a mirror compared to just 32% of Chinese students. Most Croatian scholars showed poorer oral health, with 33.1% agreeing that “My gums tend to bleed when I brush my teeth”, compared to only 5% of British students and 31% of Chinese dental students. Also, 93.7% of Croatian students reported noticing “white sticky deposits on my teeth”, while 75% of Chinese students acknowledged the same, significantly higher than the 11% of British students [30]. Croatia should enhance its prevention efforts and promote education on oral health.

We should recognize that cultural differences, attitudes, and social norms shape certain factors influencing health behaviors. Future studies should explore these relationships in more diverse populations using longitudinal designs to better understand the long-term impact of personality traits on oral health outcomes. Additionally, qualitative studies could provide deeper insights into how individuals perceive their own personality traits in relation to their health behaviors. Tailored interventions for individuals with high OCPD tendencies could optimize their strengths while mitigating risks, ultimately improving their physical and psychological well-being.

This study has several limitations that warrant consideration. Self-reported questionnaires may introduce biases related to recall accuracy or social desirability, as well as false statements. Moreover, the sample consisted exclusively of biomedical students from a single institution, limiting the generalizability of the findings to other populations or educational settings. Additionally, the cross-sectional design precludes causal conclusions about the relationship between OCPD tendencies and oral health behaviors.

## 5. Conclusions

In conclusion, this cross-sectional questionnaire-based study provides valuable insights into how personality traits influence oral health behaviors among biomedical students. The results imply that OCPD tendencies could possibly be associated with better oral hygiene practices due to traits like perfectionism and fastidiousness while they also highlight possible risks such as maladaptive over-care behaviors. However, further larger longitudinal studies are needed to address this issue.

## Figures and Tables

**Table 1:** Sociodemographic characteristics of the study sample.

Parameter	Study Sample N = 384	Lower Tendency Toward OCPD FFOCI-SF ≤ 144 N = 206	Higher Tendency toward OCPD FFOCI-SF > 144 N = 178	*p*
Gender (N, %)				
Male	112 (29.2)	73 (35.4)	39 (21.9)	0.005 *
Female	272 (70.8)	133 (64.6)	139 (78.1)	
Age (years)	23.2 ± 1.9	22.8 ± −2.0	23.4 ± 1.7	0.003 ^‡^
Economic situation (N, %)				
Lower than average	23 (6.0)	10 (4.9)	13 (7.3)	0.109 ^#^
Average	270 (70.3)	139 (67.5)	131 (73.6)	
Above average	91 (23.7)	57 (27.7)	34 (19.1)	
Study (N, %)				
Medicine	172 (44.8)	85 (40.8)	66 (44.0)	0.703 *
Dental medicine	150 (39,1)	91 (44.2)	81 (45.5)	
Pharmacy	62 (16.1)	31 (15.0)	31 (17.4)	
Study year (N, %)				
1st	42 (10.9)	30 (14.6)	12 (6.7)	0.001 *
2nd	48 (12.5)	37 (18.0)	11 (6.2)	
3rd	50 (13.0)	21 (10.2)	29 (16.3)	
4th	56 (14.6)	29 (14.1)	27 (15.2)	
5th	60 (15.6)	34 (16.5)	26 (14.6)	
6th	128 (33.3)	55 (26.7)	73 (41.0)	
Average grade	4.0 (3.8–4.2)	3.9 (3.7–4.0)	4.1 (3.8–4.2)	<0.001 ^†^
Smoking (N, %)				
Yes	118 (30.7)	72 (35.0)	46 (25.8)	
No	266 (69.3)	134 (65.0)	132 (74.2)	0.069 *
Alcohol consumption (N, %)				
Yes	222 (57.8)	139 (66.5)	85 (47.8)	0.003 *
No	162 (42.2)	69 (33.5)	93 (52.2)	

All data are presented as whole numbers (percentage), mean ± standard deviation, or median (interquartile range). * Chi-square test. ^#^ Fisher’s exact test. ^†^ Mann–Whitney U test. ^‡^ Student’s *t*-test.

**Table 2:** HU-DBI items and the study sample answers.

Parameter	I Don’t Agree	I Agree
1. I do not worry much about visiting the dentist.	102 (26.6)	282 (73.4)
2. My gum tends to bleed when I brush my teeth.	362 (94.3)	22 (5.7)
3. I worry about the color of my teeth.	220 (57.3)	164 (42.7)
4. I have noticed some white sticky deposits on my teeth.	360 (93.7)	24 (6.2)
5. I use a child sized toothbrush.	382 (99.5)	2 (0.5)
6. I think that I cannot help having false teeth when I am old.	326 (84.9)	58 (15.1)
7. I am bothered by the color of my gum.	368 (95.8)	16 (4.2)
8. I think my teeth are getting worse despite my daily brushing.	288 (75.0)	96 (25.0)
9. I brush each of my teeth carefully.	88 (22.9)	296 (77.1)
10. I have never been taught professionally how to brush.	257 (66.9)	127 (33.1)
11. I think I can clean my teeth well without using toothpaste.	319 (83.1)	65 (16.9)
12. I often check my teeth in a mirror after brushing.	78 (20.3)	306 (79.7)
13. I worry about having bad breath.	244 (63.5)	140 (36.5)
14. It is impossible to prevent gum disease with tooth brushing alone.	308 (80.2)	76 (19.8)
15. I put off going to dentist until I have a toothache.	293 (76.3)	91 (23.7)
16. I have used a dye to see how clean my teeth are.	332 (86.5)	52 (13.5)
17. I use a toothbrush which has hard bristles.	288 (75.0)	96 (25.0)
18. I do not feel I have brushed well unless I brush with hard strokes.	294 (76.6)	90 (23.4)
19. I feel I sometimes take too much time to brush my teeth.	264 (68.7)	120 (31.2)
20. I have had my dentist tell me that I brush very well.	106 (27.7)	278 (72.3)

All data are presented as whole numbers (percentage). Abbreviations: HU-DBI—The Hiroshima University Dental Behaviour Inventory.

**Table 3:** Correlation of HU-DBI subdimension with the FFOCI-SF total score.

Parameter	Study Sample N = 384	r *	*p*
Knowledge	4.0 (3.0–4.0)	0.223	<0.001
Attitudes	2.0 (2.0–2.0)	0.231	<0.001
Behavior	2.0 (1.0–2.0)	0.272	<0.001

All data are presented as median (interquartile range). Abbreviations: FFOCI-SF—Five Factor Obsessive–Compulsive Inventory—Short Form; HU-DBI—The Hiroshima University Dental Behaviour Inventory. * Spearman correlation coefficient.

**Table 4:** Correlation of FFOCI-SF subdimensions with the HU-DBI total score.

Parameter	Study Sample N = 384	r *	*p*
Excessive Worry	14.0 (12.0–16.0)	0.213	<0.001
Detached Coldness	9.0 (7.0–11.0)	−0.013	0.747
Risk-Aversion	12.0 (10.0–14.0)	0.036	0.474
Constricted	9.0 (8.0–11.0)	0.044	0.380
Inflexible	11.5 (9.0–14.0)	0.072	0.154
Dogmatism	13.0 (11.0–14.0)	0.184	0.003
Perfectionism	14.0 (12.0–16.0)	0.359	<0.001
Fastidiousness	13.0 (12.0–14.0)	0.386	<0.001
Punctiliousness	10.5 (9.0–12.0)	0.322	<0.001
Workaholism	11.0 (9.0–13.0)	0.290	<0.001
Doggedness	11.0 (9.0–14.0)	0.375	<0.001
Ruminative Deliberation	14.0 (12.0–16.0)	0.460	<0.001

All data are presented as whole numbers (percentage). Abbreviations: FFOCI-SF—Five Factor Obsessive–Compulsive Inventory—Short Form; HU-DBI—The Hiroshima University Dental Behaviour Inventory. * Spearman correlation coefficient.

## Data Availability

The research data are available upon request to the corresponding author email.
